# A Population Health and Lifestyle Survey of a Coastal City in England (Health Counts 2024): Protocol for a Cross-Sectional Study

**DOI:** 10.2196/64001

**Published:** 2025-08-21

**Authors:** Nigel Sherriff, Kate Gilchrist, Massimo Mirandola, Jörg Huber, Catherine Aicken, Kathleen Galvin, Alexandra Sawyer, Susannah Davidson, Ciara Gray, Caroline Mary Vass, Louise Knight, Carrie Llewellyn

**Affiliations:** 1 School of Education, Sport, and Health Sciences University of Brighton Falmer United Kingdom; 2 Brighton & Hove City Council Brighton United Kingdom; 3 Infectious Diseases Division Department of Diagnostics and Public Health University of Verona Verona Italy; 4 Department of Research & Knowledge Exchange University of Brighton Falmer United Kingdom; 5 Department of Primary Care & Public Health Brighton & Sussex Medical School University of Sussex Falmer United Kingdom

**Keywords:** health, survey, health inequalities, population, Health Counts 2024, public health

## Abstract

**Background:**

Health and lifestyle population surveys are important in public health to identify trends, provide data to monitor the effectiveness and reach of public health initiatives and policies, and help allocate health resources more equitably. Surveys are a methodologically robust way of examining the inequalities in health outcomes or access to resources across a number of sociodemographic groups in a defined geographic area. This particular public health survey will provide information that cannot be obtained from other sources.

**Objective:**

The aim of the study is to generate comprehensive public health–relevant data from an adult population in a defined geographic coastal area of South East England.

**Methods:**

A cross-sectional, noninterventional (observational) health and lifestyle population survey was developed using a mobile-first design approach to recruitment, with the content drawing on a previous iteration of the survey in 2012. Previous Health Counts surveys in this region have been conducted approximately every 10 years since 1992 to provide data about trends over time. Extensive rounds of consultation and testing took place between October 2023 and February 2024. The final survey comprised 102 questions structured around 13 contemporary public health-related issues in the United Kingdom. Survey distribution was carried out in 2 rounds of SMS text messaging through all general practices in Brighton and Hove involving all adults registered with a general practice and having a mobile phone who had not opted out of communications. Advertising across a range of public-facing initiatives was also used, as well as targeted outreach activities for potentially marginalized groups, for example, in public libraries and community groups. Enrollment took place between March 18, 2024, and April 28, 2024.

**Results:**

A total of 26,014 eligible people responded. Data analysis has started, and results will be reported later in 2025.

**Conclusions:**

Understanding trends in population inequalities over time as well as gaining insights into new areas for the very first time, Health Counts 2024 data can inform decision-making on strategies to improve health and reduce inequalities by local authorities in not only England and the National Health Service but also potentially across other European cities through the effective dissemination and sharing of promising practices as an integral part of evidence-based public health.

**International Registered Report Identifier (IRRID):**

DERR1-10.2196/64001

## Introduction

### Background

Coastal communities in England often face poor public health outcomes due to a combination of socioeconomic, environmental, and health care access factors [1]. Reports have demonstrated that coastal towns and cities have a higher burden of disease across a range of physical and mental health conditions. Life expectancy, healthy life expectancy, and disability-free life expectancy are often lower in coastal areas compared to inland regions [1,2]. One of the key reasons for these inequalities is the relatively higher levels of economic deprivation, which include lower income, higher unemployment, and lower economic activity. Economic deprivation is strongly correlated with health outcomes through multiplicative risk factors, such as smoking, alcohol use, access to healthy food and environments (including housing and recreational space), stress, and anxiety. Some coastal communities experience higher levels of social isolation, which is particularly pertinent among older adults and people aged <24 years [1]. Social isolation is associated with poorer mental health, such as anxiety and depression, and can also negatively affect physical health. Coastal regions more often have a high proportion of residents who are aged >65 years, which is set to increase with time [3].

The city of Brighton and Hove, in South East England, United Kingdom, has many similarities to other coastal regions; however, it has a disproportionate population of young people due to there being 2 universities. It has a diverse population and varied socioeconomic conditions; however, there are significant inequalities in health, well-being, and deprivation across different neighborhoods. Lower layer Super Output Area ranks demonstrate areas that are among the most deprived in England alongside areas that are the least deprived [4,5], making public health initiatives complex. Within Brighton and Hove, there is an inequality gap in life expectancy associated with the most and least deprived areas (10.6 y for male individuals and 6.6 y for female individuals [5]), making it important to fully understand the impact of health inequalities on outcomes [6,7].

There is a lack of granular data at a population level, especially with regard to the impact of social isolation, housing conditions, and neighborhood safety on health and well-being indices. Therefore, health and lifestyle population surveys, such as the one described in this protocol, are an important method of public health research. Their ability to generate robust information by geographical area, capacity to provide trend data, ability to focus on important new issues across the population, and ability to explore health and other inequalities across the population allow us to provide segmented data and identify inequalities across our city.

This protocol describes the development of the 2024 Brighton and Hove Health Counts Survey which was commissioned to better understand the health indices, behaviors, and health concerns of the population in a coastal city in South East England. Health Counts 2024 builds on 3 former surveys conducted in 1992 (as “Health Quest”), 2003, and 2012 (as “Health Counts”). In each case, a random sample of the population was drawn from the city. The most recent Health Counts 2012 drew a random sample of 2.5% of the population aged ≥18 years via the general practice (GP) registration database. The 2012 questionnaire covered physical and mental health, sexual health, self-harm and drug use, a range of other health topics [8], and the Office for National Statistics defined questions for a measure of happiness and personal well-being. More than a decade later, Health Counts 2024 has been commissioned by the Brighton and Hove City Council (BHCC) Public Health Team to develop a new survey that includes similar items to the previous survey (allowing tracking of inequalities over time) while also introducing changes for updates and improvements and changes to reflect local public health priorities and national public health guidance (eg, chief medical officer recommendations).

### Objectives

This paper presents the protocol for the Health Counts Survey 2024, which will allow researchers to explore the current health and well-being trends and inequalities across a defined population in a coastal region of South East England, United Kingdom. The overarching aim of this study is to generate comprehensive public health–relevant data from an adult population in a defined geographic area of South East England. Specifically, the research objectives are to (1) obtain a range of data on health and well-being, health behavior, lifestyles, and broader social determinants of health, aiming for a sample that is broadly representative of the city of interest’s adult population; (2) provide comparable data to previous population surveys of the city of interest, enabling changes to public health in the city to be tracked over time; and (3) allow population-level inequalities to be explored both cross-sectionally and longitudinally.

## Methods

### Design

This is a cross-sectional, noninterventional (observational) health and lifestyle population survey.

### Study Population

The study population for Health Counts 2024 was defined as anyone aged ≥18 years residing either temporarily or permanently or registered with a GP within the city council boundaries. There was no sample size calculation for this convenience survey. As of the census of 2021, the population of Brighton and Hove was 277,200 (141,600 female and 135,600 male individuals). The total adult population of Brighton and Hove is estimated at 240,000.

### Inclusion and Exclusion Criteria

The inclusion and exclusion criteria are presented in Textbox 1.

**Inclusion and exclusion criteria.**

**Inclusion criteria**

- Aged ≥18 years
- Currently living in Brighton and Hove permanently or temporarily (eg, students, travelers, refugees, and asylum seekers)
- Living elsewhere but registered at a general practice located within the city of Brighton and Hove

**Exclusion criteria**

- Aged <18 years
- Responses indicating residence outside of the city of Brighton and Hove and that they are not registered with a general practice in the city of Brighton and Hove

### Design of the Questionnaire and Testing

The initial design for Health Counts 2024 was based on 3 previous versions of the survey (Health Quest 1992, Health Counts 2003, and Health Counts 2012). Each of these former surveys was designed to be paper based and self-completed. In the development of Health Counts 2024, the following considerations were discussed:

1. Retaining key topics from previous Health Counts and Health Quest surveys to be able to examine change over time versus replacing or adding new emerging public health topics
2. Keeping questions as close as possible to Health Counts 2012 for comparability versus adjusting questions to improve measurements, modernize language, or take into account changes in licensing of some standardized scales (eg, replacing the 12-Item Short Form Health Survey or 36-Item Short Form Health Survey with the license-free Veterans RAND 12-Item Health Survey [VR-12])
3. Providing an increased number of response options to better reflect people’s experience and increase data specificity versus fewer response options (required for a mobile-first approach), which potentially increases ease of completion and later data management
4. Balancing having a longer survey capturing more factors of interest but with potentially increased dropout and partial completion with retaining a representative sample, especially those from underresourced groups

An initial critical review of the previous Health Counts 2012 survey was conducted by the study team (the authors) of Health Counts 2024 in February 2023. This was to identify which questions could potentially be removed, adjusted, or kept. This process considered aspects such as wording and phrasing (eg, outdated language and terminology), questions about topics where good-quality recent local data were already available, emerging topics of interest, and backward comparability with previous surveys to allow for analyses of trends over time.

Following this initial review, an in-person consultation event was held with the BHCC Public Health Team, comprising colleagues with expertise in the areas covered by the survey, to discuss and agree on the items to bring forward from the 2012 version as well as identify additional topics and additional outcome measures for potential inclusion. During this consultation, issues discussed also included the length of the survey (ideally to be completed within a maximum target time of 20 min), balance of topics, acceptability to different populations (eg, items on suicide ideation), as well as which validated scales should be retained and adjusted or new ones included (eg, Global Physical Activity Questionnaire) [9].

After the BHCC consultation, a questionnaire draft was created with the questions structured in conceptual order and arranged in 10 groups: about you (demographics), general health, mental health and well-being (including self-harm and suicide), physical health, diet, smoking and vaping, alcohol, drugs, sexual health, and local area and neighborhood. Information was provided on the source of each item, with any new topics and items highlighted in red. This second consultation was then held, this time by email, with the BHCC Public Health Team; local community and voluntary organizations; and other stakeholders, including representatives from the wider partnership, such as the National Health Service (NHS) Sussex, statisticians, and a wide array of experts in public health population survey design. As a result of this second round of consultation, numerous changes to existing questions were made, with decisions to include or exclude or change items accordingly.

Parallel to these consultation events, several platforms to host the survey were tested. Requirements were to be a secure, General Data Protection Regulation (GDPR)–compliant online platform allowing routing between questions, attractive presentation of the survey, optimization for mobile and other devices, and a “backend” that could be programmed to minimize data cleaning and recoding effort. Three platforms were trialed (in order), including REDcap (Research Electronic Data Capture; Vanderbilt University), Joint Information Systems Committee online surveys (version 2), and Qualtrics (Qualtrics International Inc). Preliminary test versions of sections of the questionnaire (eg, demographic questions) were entered into each platform and prepiloted with people known to the study team of Health Counts 2024. Personal invitations were sent to academic colleagues at the authors’ own institutions as well as practitioners from the local government authority’s public health team. The purpose of these prepilots was to explore the limits of the software, including mobile design “friendliness”; investigate functionality around routing; and provide opportunistic possibilities to test out discrete sections of the questionnaire as they became available, checking for acceptability, completeness, phrasing, and comprehension.

Further pretesting during a planned period of public patient involvement and engagement (PPIE) activities also garnered feedback on the look and feel of the survey on each of these platforms as well as ease of use on various devices (eg, Apple vs Android mobile operating systems on mobile devices, tablets, and PCs). PPIE activities were conducted during September 2023 and October 2023 by Healthwatch Brighton and Hove (a community interest company that represents the views of the people who use health and social care services in the city) with diverse communities, including populations that are often under-resourced and absent from research (eg, older people; those at risk of digital exclusion; homeless people; and Gypsy, Roma, and Traveller communities). As part of these activities, Healthwatch Brighton and Hove was asked to engage with 10 to 15 people individually or in a group setting to include those whose first language was English, people who do not classify themselves as White British (to include Gypsy, Roma, and Traveller communities), a range of gender identities, and people aged >18 years. On the basis of this feedback from 13 people (n=9, 69% individuals and 1 group of n=4, 31% individuals) as well as other practical considerations (eg, functionality), Qualtrics was confirmed as the platform of choice, which includes a dedicated mobile design build optimized for mobile-first design.

A third and final consultation event was held among the research team partners (universities, local authority, and Healthwatch Brighton and Hove) in October 2023 to finalize the questionnaire items. A first full draft of the Health Counts 2024 questionnaire was then developed, representing a compromise between inclusivity and brevity. While prioritizing a mobile-first approach and respondent experience, we nevertheless aimed to make the survey as useful as possible to the largest number of stakeholders.

During November 2023, a second full draft of the survey was developed, which included the proposed introductory text, exit page texts, page and topic headings, details of the prize draw, support organizations, and any additional instructions to participants. At this point, this document was used as the basis to translate into a full online version using Qualtrics. From this point, all pretesting, PPIE, and piloting were conducted using the online version only.

### Final Testing and Online Piloting Checks

Given extensive pretesting, the main aim of the pilot in February 2024 was to test the survey in its most complete form and provide sufficient data for validity checking of particular questions. The survey was kept open online for 1 week to collect responses from participants outside of Brighton and Hove who would not be eligible to take part in the final survey. In total, 50 responses were received during the piloting, and no major problems were evident. The piloting highlighted some minor issues that needed attention, including some routing errors, typos, and opportunities to reduce “clutter” in line with the design approach (eg, removal of brackets, full stops, and bold and italics). The pilot dataset was downloaded using SPSS (IBM Corp) and imported into Stata (StataCorp) to generate what would become the syntax during the survey launch itself to allow the team to monitor completions on a daily and weekly basis, thus informing proactive promotion and marketing of the survey across the city.

### Final Questionnaire Content

The final version of the questionnaire comprised 102 questions structured around 13 contemporary public health-related issues in the United Kingdom (Table 1). A copy of the final questionnaire is available in Multimedia Appendix 1.

**Table 1:** Final questionnaire content of Health Counts 2024.

Categories	Items
Survey eligibility check	Items assessing eligibility of age, postcode, and general practice
Sociodemographics	Items about participants’ age, disability, ethnicity, marital, civil, and relationship status, religion, gender identity, sexual orientation, carer status, and armed forces status—including family, employment and education, household, and refugee and asylum seeker status
General health	Items assessing happiness, general health status, as well as long-term conditions and disabilities and their impact Includes items from the VR-12^a^ [10]
Physical health	Items assessing the impact of health on daily living and activities and the impact of pain, falls, and active and sedentary behaviors Includes items from the VR-12 standardized scale, the IPAQ^b^ [11], and the SALS^c^ [12], measuring adult physical activity levels
Mental health and well-being	Items assessing the impact of mental health on daily living and activities, energy fatigue, social functioning, anxiety, depression, self-harm, suicidal ideation, and suicide attempt Includes items from the VR-12 [10]
Smoking and vaping	Items assessing the use of tobacco (smoking and other), addiction, quit attempts, household smoking, and vaping status
Alcohol consumption	Items assessing frequency of alcohol use, units consumed, binge drinking, and alcohol reduction attempts. Includes items from the AUDIT^d^ [13]
Gambling	Items assessing gambling participation and gambling impact (own and others)
Drugs	Items assessing recent drug use and drug type
Sexual health	Items assessing the number of partners last year, the number of new partners, new partner type, condom use (new partners only), HIV testing (ever and recency), and preexposure prophylaxis to prevent HIV awareness and use
Diet, height, and teeth	Items assessing fruit and vegetable consumption (“5 a day”), perceived weight status, BMI (height and weight) [14], teeth cleaning frequency, source of dental support, dentist visit frequency, and reasons for attendance or nonattendance
Housing and the cost of living	Items assessing housing status, housing condition, and the cost-of-living impact
Local area	Items assessing local area satisfaction, neighborhood belonging, neighbor contact, social support, access to nature (frequency and type), local area safety (day and night), fear of crime, and experienced harassment

^a^VR-12: Veterans RAND 12-Item Health Survey.

^b^IPAQ: International Physical Activity Questionnaire.

^c^SALS: Short Active Lives Survey.

^d^AUDIT: Alcohol Use Disorders Identification Test.

### Conceptual Approach to Methodology

The survey was underpinned conceptually by theoretical ideas coming from the theory of planned behavior [15] and other conceptual frameworks, such as the health belief model [16], social cognitive theory [17], and ecological models such as the one by Bronfenbenner [18]. Together, these frameworks emphasize how behavior and lifestyle are influenced by, for example, complex interactions among personal, social, cultural, environmental, and behavioral factors. Furthermore, a mobile-first design approach was also used to underpin the survey development from the outset. This approach goes beyond simply making a survey available online and accessible by any device. Rather, mobile-first research is designed from the bottom up to be conducted on a mobile phone. Such a design entails paying attention to how mobiles are used in day-to-day life and creating the right conditions for participants to engage and complete the survey. For example, the survey should be short, simple, and easy to navigate (minimizing things such as sliders and drop-down boxes that require additional actions by the user, minimizing open text responses, minimizing any horizontal scrolling, keeping instructions short and concise, removing unnecessary wording, being visually engaging, and using short response scales). Therefore, this mobile-first thinking informed all aspects of the Health Counts 2024 design, being balanced against the requirements of a degree of backward comparability with previous paper-based health counts questionnaires.

### Procedure and Recruitment

Health Counts 2024 opened at 7 AM on Monday, March 18, 2024, and ran for 6 weeks until April 28, 2024, 8 PM. The survey was hosted on the University of Brighton landing page [19], containing a call-to-action button to the survey as well as detailed participation information and details of relevant support organizations on issues covered by the survey (eg, sexual health, smoking, drug use, and mental health support).

Previous Health Counts surveys (1992, 2003, and 2012) were conducted as postal self-completion surveys, sent to a random 2% to 2.5% sample of adults aged >18 years registered in GP. The most recent data from NHS Digital [20] and the Office for National Statistics [21] indicate that the number of people registered with GPs in England is higher than ONS population estimates and for Brighton and Hove, where this survey was conducted, the difference is estimated to be 4% to 9% higher than the resident population for various reasons [22]. Due to this relatively high level of registration among adult populations, recruitment through GP services was selected as the main method of enrollment. For the 2012 survey, public health was located within the NHS; therefore, samples could be drawn using the Primary Care Support Services. This meant that patient contact details could be provided directly to researchers, who could then invite them to participate. With the introduction of GDPR, there was no option to generate a random sample for the 2024 survey. Therefore, in discussion with a wide range of stakeholders and partners (eg, the NHS information governance team, the data protection officer for GP, the GP Practice Managers’ Forum, the local medical committee, and the Brighton and Hove Health and Care Partnership), a population approach was adopted whereby each GP in the city would be asked to send SMS text messages to eligible registered patients with an invitation to complete the survey. In these discussions, the Practice Managers’ Forum suggested that, as there was no way for practices to identify a random sample of patients, all patients aged >18 years should be invited to participate. Such an approach would mean GPs would be able to reach most of the local population (estimated at approximately 98%). This process is compliant with the Data Protection Act 2018, which is the United Kingdom’s implementation of the GDPR, provided practices first run the patient list through the Message Exchange for Social Care and Health to remove any patients with a National Data Opt Out (a service that allows patients to opt out of their confidential patient information used for research and planning). We also engaged with the National Institute for Health and Care Research Clinical Research Network to confirm that contacting patients in this way is reasonable and appropriate in terms of offering patients the opportunity to participate in health-related research.

Between November 2023 and March 2024, the Health Counts team liaised either directly with GPs or via the Brighton and Hove Federation to request them to (1) send 2 SMS text messages to eligible patients with a link to the survey (1 initial invitation and 1 reminder) and (2) promote the survey to their patients through the practice (eg, link or QR code on the practice website, electronic poster on a digital screen, and social media channels). Practices were reimbursed by the public health grant for the time taken to run searches and message patients as well as the cost of SMS text messages being paid for.

Practices were asked to send the first text on the first day of the survey launch (March 18, 2024), ideally either between 7 AM and 10 AM or 2 PM and 8 PM. This timing was proposed to maximize potential responses based on the literature suggesting that these are the times patients may be more likely to complete the survey. The second text was requested to be sent on Monday, April 15, 2024, either between 7 AM and 10 AM or 2 PM and 8 PM. These times were indicative, and some practices opted to send messages to batches of patients across the relevant weeks rather than all in one go to minimize potential disruption to reception staff with potential queries.

### Community Engagement and Supported Completion

To maximize the potential to engage and support those often underrepresented in research or those who might have challenges in accessing and completing the survey (eg, homeless or not registered with a GP in the city), targeted outreach activities were conducted. Outreach comprised collaborating with community organizations, voluntary sector groups, and charities to share information on their websites and social media platforms and meeting community groups to (1) explain the purpose and nature of the survey and (2) assist people to access the survey by providing digital devices (eg, tablets) and, where necessary, a hard copy or supported completion by a member of the research team, if required. Organizations that already support groups in the following areas were approached directly: young people; learning disability; mental health; sexual health; homelessness; women’s support; gender; minority and ethnic communities, including Gypsy, Roma, and Traveller communities; and older people. Two full-day drop-in sessions were also conducted in a local library to support general community engagement. In addition, community outreach by 2 local football clubs facilitated awareness raising at 2 match days. A wide range of organizations supported awareness raising, disseminated information through their own social media platforms and digital networks, and facilitated public engagement and hands-on support with the survey. In addition, other community services used their own digital platforms and digital screens to raise awareness of the survey for their service users. This complemented the general marketing strategy described subsequently by targeting specific groups in the population of Brighton and Hove. Where the research team met with local community groups or was present in the library, support to engage with the survey through a digital device or hard copy survey was offered, or “hands-on” support was offered for individuals to use their own digital device, such as a mobile phone. The intention was to assist individuals in completing the survey themselves and enhance access, address concerns, and support informed consent.

### Promotion of the Survey

To help raise awareness about Health Counts 2024 for those receiving SMS text messages from their GP as well as wider opportunities to engage through the outreach activities or independently of these routes (eg, directly via QR codes), a comprehensive marketing and communication strategy was developed in collaboration with an external design contractor to provide an attractive, coherent, and augmented visual identity. This strategy included both paid and unpaid promotional activities through the partner organizations as well as a paid social media campaign across the major platforms during the survey launch window. Daily statistical monitoring of the survey completions allowed promotion to be adjusted throughout the campaign to target, for example, those less likely to complete the survey (eg, young people and men) or geographical areas where completion rates differed substantially. The range of promotional activities and their locations were diverse, including, for example, pull-up banners and LED and plasma screens in public spaces (such as libraries, other public buildings, and council offices), posters, radio advertisements, mailers and newsletters, university student alerts, web banners and email signatures, digital bus stop promotions, and hard-copy flyers and posters as well as the use of community apps, forums, and newsletter articles.

### Data Cleaning and Analyses

Data were cleaned and checked for eligibility before starting analysis. Attrition, nonresponse, and missing data were examined and dealt with at the appropriate level of analysis. A detailed codebook describing each of the items and response frameworks was used to guide the scoring of each item and construct. For validated scales (eg, the VR-12 [10], International Physical Activity Questionnaire [11], Global Physical Activity Questionnaire [9], and Alcohol Use Disorders Identification Test C [13]), scores will be created following standardized instructions, including instructions for dealing with missing values in the case of the VR-12.

Data analyses will be primarily exploratory and descriptive initially. Descriptive findings will be reported as means and SDs for continuous variables and numbers and percentages for categorical variables. To ensure internal consistency of scales, reliability will be checked with Cronbach α. Where feasible and appropriate, estimates will be compared to values from national surveys, such as the UK Census 2021 and other annual population surveys (eg, the People and Nature Survey for England from April 2023 to March 2024) [23], covering similar time periods. In addition, and again where possible, comparisons will also be made against data generated from previous iterations of the Health Counts survey (1992, 2003, and 2012).

Descriptive analyses will be run in SPSS (version 29.02.0) and include bivariate analysis, including chi-square tests (or Fisher exact test when appropriate) and Mann-Whitney *U* tests, which will be used to determine significant differences between groups for categorical variables, including demographics. Kruskal-Wallis tests will be used for continuous variables. CIs will be reported where relevant.

Given the likely deviations of the sample composition from the population, we will statistically weight our findings accordingly. This will make the data more representative of the population they are designed to represent, that is, Brighton and Hove as a whole. Apart from this form of frequency weighting, we will consider using a poststratification approach to sampling weights, which will provide improved estimates of measures of variation, including SEs. The weights for Health Counts 2024 will be calculated with the help of census data from 2021 and will include age, (binary) sex, and Index of Multiple Deprivation quintiles [24].

While most questions in the survey were closed, a limited number did allow open-ended responses so respondents could elaborate or “specify” if they felt the standard response options did not apply to them. To analyze these qualitative data, we expect to apply content analysis to the verbatim responses given to the open-ended questions. This will ensure text can be systematically categorized to meaningfully quantify the qualitative (descriptive) responses.

### Ethical Considerations

#### Overview

Initial approval for recruitment via GPs (NHS recruitment pathway) was granted by the University of Brighton Pre-Sponsorship Approval Panel and the University of Brighton Sponsorship Approval Panel (2023-11973). The ethics approval was granted by the NHS Health Research Authority (23/LO/0825) and the London—Bromley Research Ethics Committee (23/LO/0825). In this study, GPs were acting as participant identification centers, and each participating GP signed a participant identification center agreement outlining the responsibilities of the practice and study sponsor. The ethics approval for the community enrollment and supported completion pathway was granted by the University of Brighton Cross-School Research Ethics Committee C (2023-12553).

#### Confidentiality

No personal data (such as names, addresses, and dates of birth) were collected from participants. The survey was completely anonymous; no IP addresses were stored or downloaded, and no information regarding the origin of the “click” was collected. No cookies were installed on the potential participant’s computer or device. There was no compensation provided to participants.

#### Informed Opt-In Consent

Respondents who received an SMS text message invitation to participate in Health Counts 2024 from their GP had to click on the link sent to them in the message, which took them to the survey landing page. This page contained an action button to the survey as well as detailed participation information and details of relevant support organizations on issues covered by the survey (eg, sexual health, smoking, and drug use). The information provided included information about the project, confidentiality of the survey findings, and an outline of what participants were required to do and how long it would take them to complete the questions. A statement was provided regarding data protection, including confidentiality and anonymity. Potential participants were asked to click on a box to confirm that they had (1) read and understood the participant information before proceeding; (2) understood that the survey was voluntary and anonymous and their answers would not be traceable to them; and (3) agreed to take part in the survey.

## Results

### Recruitment

Participant enrollment took place between March 18, 2024, and April 28, 2024, with 26,014 eligible respondents. The response rate to the survey is estimated to be 11.2% of the resident population aged ≥18 years based on the July 2024 Office for National Statistics’ midyear population estimates release for 2023 (232,971 adults). As expected, there were some incomplete surveys submitted; of the 26,014 submissions, 16,729 (64.31%) were complete, giving a final weighted sample and response rate of 7.2% of the resident population aged ≥18 years. Data analysis will be completed in 2025, with results expected by the end of 2025.

### Analysis

Data analysis for the primary outcome paper, along with planned secondary analyses, is currently underway, with the intention of disseminating study findings in peer-reviewed journals. Findings from the analyses will also be disseminated to voluntary and community sector organizations (including the public) through specific accessible summary reports and accompanying presentations as well as representatives of the local government and the NHS. The planning, preparation, and submission of publications will follow the Health Counts 2024 Editorial Board policy, which draws on international guidance for authorship, including the International Committee of Medical Journal Editors [25] criteria. In addition, findings will also be shared with a broad scientific audience through presentations at local, national, and international conferences.

## Discussion

### Anticipated Findings

Health and lifestyle population surveys are essential in public health to identify trends, provide data to monitor the effectiveness and reach of public health initiatives and policies, and help allocate health resources more equitably. To our knowledge, this study is one of the first internet-based self-completion questionnaire surveys that, in partnership with the local authority, the NHS, primary care, and other partners, has the potential to transform understandings of the health and well-being of the residents of the city of Brighton and Hove. It is expected that results will (in some cases) be able to provide an indication of inequalities over time by comparing with previous versions of the survey and also in other important emerging areas, such as gambling, vaping, community safety, harassment, and hate crime, drawing attention to issues where data are less available. Indeed, by understanding better historical inequalities as well as new emerging public health areas of interest, including threats, the findings are also expected to generate important insights across different local authority departments directly relevant to public health and its social and wider determinants (eg, housing, environment, and transport).

Health Counts 2024 survey data will also be important in helping researchers identify population-level health inequalities and also critically inform decisions about future health and well-being strategies and services to better meet residents’ needs. It will thus provide a wealth of information that cannot currently be obtained from other sources and may be of particular relevance for government bodies regionally and nationally as well as in wider international settings (eg, such as the World Health Organization Healthy Cities Network).

There are some limitations to this study, including the cross-sectional nature of the design methodology. Although this survey includes some items linked to the 3 previous iterations of the survey, allowing for the analysis of inequalities over time to be conducted, new items and topics introduced for the first time in Health Counts 2024 (including gambling-related harms, suicidal thoughts and attempts, harassment and hate crime, alongside access to nature and the natural environment) limit the ability to make causal inferences or assess temporal relationships about these items and topics. Moreover, as health and lifestyle surveys cover a variety of issues that are commonly stigmatized and include sensitive topics (eg, alcohol and drug use, physical activity, weight, suicide and suicide ideation, and the fear of crime), there is potential for information bias in the form of social desirability. However, it is also likely that due to the anonymous nature of the survey, the fact that the primary invitation to participate was sent to participants through GP in the city, and also the known fact that the public trust in the NHS and primary care is high, it is possible that social desirability concerns may have been suitably mitigated.

### Conclusions

The Health Counts 2024 survey was designed to generate comprehensive public health data from an adult population in a coastal area of South East England. It is expected that our results can be used to inform future public health and other intersectoral policies and interventional campaigns and health care provider–based interventions that introduce new models of care for city residents. Through better understanding the trends in population inequalities over time (where data allow) as well as gaining insights into new areas for the first time, it is hoped that the data can inform decision-making on strategies to improve health and reduce inequalities by local authorities in not only England and the NHS but also potentially across other European cities through effective dissemination and sharing of promising practices as an integral part of evidence-based public health.

## Appendix

Multimedia Appendix 1 Health Counts 2024 questionnaire.

## Abbreviations

BHCC: Brighton and Hove City Council
GDPR: General Data Protection Regulation
GP: general practice
NHS: National Health Service
PPIE: public patient involvement and engagement
REDCap: Research Electronic Data Capture
VR-12: Veterans RAND 12-Item Health Survey
