# Supplementary material for: A Population Health and Lifestyle Survey of a Coastal City in England (Health Counts 2024): Protocol for a Cross-Sectional Study
Source: JMIR Res Protoc. 2025 Aug 21;14:e64001. doi: 10.2196/64001 (PMC12411788; doi:10.2196/64001)
Supplement: Multimedia Appendix 1 [file resprot_v14i1e64001_app1.pdf]

Health Counts 2024 Questionnaire – FINAL

On behalf of the Health Counts 2024 Study Team:

University of Brighton (UoB)
Nigel Sherriff, Catherine Aicken, Jorg Huber, Massimo Mirandola, Kate Galvin,
Alexandra Sawyer, Susannah Davidson, Ciara Gray, Shemane Murtagh

Brighton & Hove City Council (BHCC)
Kate Gilchrist, Caroline Vass, Louise Knight

Brighton & Sussex Medical School (BSMS)
Carrie Llewellyn

Suggested citation:

Sherriff, N.S., Mirandola, M., Huber, J., Aicken, C., Galvin, K., Sawyer, A., Davidson, S.L., Gray, C., Vass, C., Llewellyn, C.D., Knight, L. Gilchrist, K. (2024). Health Counts 2024 Questionnaire. Brighton: University of Brighton.

Health Counts 2024 is funded by Brighton & Hove City Council Public Health Department

Health Counts 2024 Questionnaire – FINAL

<the following survey is a full print version produced by Qualtrics. It includes full routing details and other items which will not be visible to respondents. Images and some aesthetic features are not included>

Survey Flow

Standard: Welcome & eligibility (2 Questions)

Branch: New Branch
If
If Device Type Is Mobile

Embedded Data
Mobile = 1

Branch: New Branch
If
If Device Type Is iPhone

Embedded Data
iPhone = 1

Branch: New Branch
If
If Device Type Is Not Mobile

Embedded Data
Mobile = 0

Branch: New Branch
If
If Consent No Is Selected

End Survey: Advanced

Standard: Eligibility (3 Questions)
Standard: You and your household (3 Questions)
Standard: A bit about you... (6 Questions)
Standard: General health (5 Questions)
Standard: Physical health (9 Questions)
Standard: Mental health and wellbeing (8 Questions)
Standard: Smoking & vaping (9 Questions)
Standard: Alcohol consumption (4 Questions)
Standard: Gambling (5 Questions)
Standard: Drugs (2 Questions)
Standard: Sexual health (8 Questions)
Standard: Diet, height and teeth (10 Questions)
Standard: Housing and cost of living (3 Questions)
Standard: Your local area (9 Questions)
Standard: Some final questions about you... (17 Questions)
Standard: And finally... (1 Questions [prize draw entry])

Health Counts 2024 is funded by Brighton & Hove City Council Public Health Department

Start of Block: Welcome & eligibility

Welcome to Health Counts 2024 Health Counts 2024 is a health and lifestyles survey that runs every ten years. Your help ensures we can better address the health and social care needs of the people of Brighton & Hove. It takes about 15 minutes to complete.

What does the survey cover? It asks about the health and lifestyle issues people in the city experience. This helps inform decisions about what services might be needed. Some questions may feel sensitive (e.g. sexual health, self-harm). Contact details for support organisations can be found in the survey and here. If you do not want to answer a question, you can skip it or answer 'prefer not to say'.

Is it private? It is anonymous. Your answers cannot be traced back to you.

Who is running the survey? The University of Brighton with Healthwatch Brighton & Hove, NHS Sussex, Brighton & Hove Federation, and Brighton & Sussex Medical School. Health Counts 2024 is funded by Brighton & Hove City Council Public Health team.

Do I have to take part? The survey is voluntary.

Prize draw: You can enter a competition to win a £100 voucher. Enter your email or contact details at the end of the survey. These details are *not* linked to your responses, are stored separately until draw completion, and then deleted.

INVITATION - Please complete if you are:

- Aged 18 or older, **and**
- Currently living in Brighton & Hove permanently or temporarily (e.g. students, travellers, refugees), or
- Living elsewhere but registered with a GP in Brighton & Hove

CONSENT - By clicking '**YES**' to the next question, you agree to participate in the survey and declare:

- I have read and understood the information above
- I understand the survey is voluntary, anonymous, and my answers will not be traceable to me
- I agree to take part in the survey

[forced response]
I agree to take part in the survey

○ Yes

○ No

End of Block: Welcome & eligibility

Start of Block: Eligibility

[response requirements >18<110]
[forced response]

How old are you?

____

Please provide your postcode adding a space in the middle e.g. BN1 9PH
[forced response]

____

What is the name of your GP practice?

Responses are confidential and will not be shared with your GP
[forced response]

- I am not registered with a GP
- Allied Medical Practice (School House Surgery or Church Surgery)
- Arch Health CiC
- Ardingly Court Surgery
- Beaconsfield Medical Practice
- Brighton Station Health Centre (Practice Plus)
- Broadway Surgery
- Carden Surgery
- Charter Medical Centre
- Hove Medical Centre
- Links Road Surgery
- Mile Oak Medical Centre
- Montpelier Surgery
- Park Crescent Health Centre
- Pavilion Surgery
- Portslade Health Centre
- Preston Park Surgery
- Regency Surgery
- Saltdean and Rottingdean Medical Practice
- Seven Dials Medical Centre
- Ship Street Surgery
- St. Peter's Medical Centre
- Stanford Medical Centre
- The Avenue Surgery
- The Haven Practice

Health Counts 2024 is funded by Brighton & Hove City Council Public Health Department

- Trinity Medical Centre
- University of Sussex Health Centre
- Warmdene Surgery
- WellBN (Brunswick, Burwash or Benfield)
- Wellsbourne Healthcare CIC
- Wish Park Surgery
- Woodingdean Medical Centre
- Other - my GP practice is not listed
- I prefer not to say
- I don't know

End of Block: Eligibility

Start of Block: You and your household

We would like to ask about your **household**, **relationships**, and **employment/education** status. Please answer every question. If unsure, just give the best answer you can.

How many **adults** including yourself live in your household?____

Do **children** under 18yrs live in your household?

- Yes
- No

Display This Question:
If Children in household = Yes

[constant sum function]

Please give the number of children in each age group:
0-4 yrs : _______
5-15 yrs : _______
16-17 yrs : _______
Total : ________

End of Block: You and your household

Health Counts 2024 is funded by Brighton & Hove City Council Public Health Department

Start of Block: A bit about you...

Are you in a relationship?

- ◯ Yes, living together
- ◯ Yes, not living together
- ◯ No

What is your legal marital or registered civil partnership status? (Select one)

- ◯ Never married and never registered in a civil partnership
- ◯ Married
- ◯ In a registered civil partnership
- ◯ Separated but still legally married
- ◯ Separated but still legally in a civil partnership
- ◯ Divorced
- ◯ Formerly in a civil partnership which is now legally dissolved
- ◯ Widowed
- ◯ A surviving member of a legally registered civil partnership
- ◯ Prefer not to say

Health Counts 2024 is funded by Brighton & Hove City Council Public Health Department

Which of the following describes what you were doing over the last 7 days? (Tick all that apply)

- ☐ I am in work/employment
- ☐ I am in education or training
- ☐ I am retired
- ☐ I am unemployed or not in work
- ☐ Looking after home or family
- ☐ Long term sick or disabled
- ☐ ⊗[1]Other (please specify) ____

Display This Question:
If Status last 7 days = I am in work/employment

In the last 7 days were you...?

- ○ Employed full-time (more than 30 hours per week)
- ○ Employed part-time (30 hours per week or less)
- ○ Employed part-time AND self-employed/freelance
- ○ Self-employed or freelance
- ○ Other (please specify) ____

Display This Question:
If Status last 7 days = I am in education or training

Which of the following best describes your situation?

- ○ Full-time student
- ○ Part-time student
- ○ Doing an apprenticeship
- ○ Other form of education or training (please specify) ____

---

1 ⊗ = Exclusive answer

Health Counts 2024 is funded by Brighton & Hove City Council Public Health Department

What is your highest educational qualification so far?

- ◯ No educational qualifications
- ◯ GCSEs/O-levels
- ◯ A-levels/AS level/Higher school certificate
- ◯ NVQ levels 1-3
- ◯ NVQ levels 4-5/HNC/HND
- ◯ First degree (e.g. BA or BSc)
- ◯ Higher degree (Masters or PhD) or other post-graduate qualification
- ◯ Other (please specify) ____

End of Block: A bit about you...

Start of Block: General health

This section asks about your **health in general**. Please answer every question. If unsure, just give the best answer you can.

Overall, how happy did you feel yesterday? On a scale of 0-10 where 0 is 'not at all happy' and 10 is 'completely happy' (Move the slider to select)

	Not happy	Completely happy
	0 1 2 3 4 5 6 7 8 9 10	

In general, would you say your health is...

- ◯ Excellent
- ◯ Very good
- ◯ Good
- ◯ Fair
- ◯ Poor

Health Counts 2024 is funded by Brighton & Hove City Council Public Health Department

Do you have any physical or mental health conditions or illnesses lasting or expected to last 12 months or more?

◯ Yes

◯ No

Display This Question:
If Disability = Yes

Tick all that apply

☐ Autism/autistic spectrum disorder or condition

☐ Developmental (e.g. affecting motor, cognitive, social, language)

☐ Hearing (e.g. D/deaf, partial loss)

☐ Learning disability

☐ Learning difference (e.g. dyslexia, dyspraxia, ADHD)

☐ Long-term physical illness/health condition (e.g. cancer, diabetes)

☐ Mental health difference / condition (e.g. depression, schizophrenia)

☐ Physical difference (e.g. condition limiting basic physical activities)

☐ Sight (e.g. blindness, partial sight loss)

☐ Speech and language

☐ Visible difference with a disabling and/or discriminatory impact

☐ Something else (please specify) ____

☐ ⊗Prefer not to say

Health Counts 2024 is funded by Brighton & Hove City Council Public Health Department

Display This Question:
If Disability = Yes

Do any of these conditions or illnesses reduce your ability to carry out day-to-day activities?

Day-to-day activities are things people do on a regular or daily basis e.g. washing, walking, reading, writing

- ◯ Yes, a lot
- ◯ Yes, a little
- ◯ No
- ◯ Prefer not to say

End of Block: General health

Start of Block: Physical health

This section asks about your **physical health**, how you feel and how well you are able to do your usual activities.

The following is a list of activities you might do in a typical day. Does your health now limit you in these activities? If so, how much?

	Yes, limited a lot	Yes, limited a little	No, not limited at all
Moderate activities, such as hoovering, swimming or playing table tennis	◯	◯	◯
Climbing several flights of stairs	◯	◯	◯

During the past 4 weeks, have you had any of the following problems with your work or other regular daily activities as a result of your physical health?

	None of the time	A little of the time	Some of the time	A lot of the time	All of the time
Accomplished less than you would like	◯	◯	◯	◯	◯
Were limited in the kind of activities you could do	◯	◯	◯	◯	◯

During the past 4 weeks, how much did pain interfere with your normal work (including both work outside the home and housework)?

- ◯ Not at all
- ◯ A little bit
- ◯ Moderately
- ◯ Quite a bit
- ◯ Extremely

Health Counts 2024 is funded by Brighton & Hove City Council Public Health Department

Have you had a fall in the past year?

Here we mean any time you have unintentionally come to rest on the ground or floor whether or not you were injured.

- ◯ No
- ◯ Yes, one fall
- ◯ Yes, more than one fall

During the last 7 days, approximately how much **time** did you spend sitting on a weekday?

Include time spent at work, at home, studying, and during leisure time. This may include time spent sitting at a desk, visiting friends, reading, or sitting or lying down to watch TV.

- ◯ Hours per day ____

In the past 7 days, have you done any sport, fitness activity (e.g. gym or fitness class), or dance?

- ◯ Yes
- ◯ No

Display This Question:
If Active behaviour = Yes

Was the effort you put into doing sport, fitness activities, or dance *usually* enough to raise your breathing rate?

- ◯ Yes
- ◯ No

Display This Question:
If Active behaviour = Yes

On how many days did you do a sport, fitness activity, or dance?
(Move the slider to select)

	1	2	3	4	5	6	7
Number of days							

Display This Question:
If Days of active behaviour [Number of days] >= 1

How much time did you *usually* spend doing sport, fitness activities, or dance on each day that you did the activity?

Hours per day	--------------
Minutes per day	--------------

End of Block: Physical health

NHS Sussex · healthwatch Brighton and Hove Your health and social care champion · University of Brighton · brighton and sussex medical school · Brighton and Hove Federation Primary Care for our local community · Brighton & Hove City Council

Health Counts 2024 is funded by Brighton & Hove City Council Public Health Department

Start of Block: Mental health and wellbeing

We would now like to ask you questions about your wellbeing. Please answer every question. If unsure, just give the best answer you can.

During the past 4 weeks, have you had any of the following problems with your work or other regular daily activities as a result of any emotional problems (such as feeling depressed or anxious)?

	No, none of the time	Yes, a little of the time	Yes, some the time	Yes, most of the time	Yes, all of the time
Accomplished less than you would like	○	○	○	○	○
Didn't do work or other activities as carefully as usual	○	○	○	○	○

These questions are about how you feel and how things have been with you during the past 4 weeks. For each question, please give the one answer that comes closest to the way you have been feeling. How much of the time during the past 4 weeks...

	All of the time	Most of the time	A good bit of the time	Some of the time	A little bit of the time	None of the time
Have you felt calm and peaceful?	○	○	○	○	○	○
Did you have a lot of energy?	○	○	○	○	○	○
Have you felt downhearted?	○	○	○	○	○	○

During the past 4 weeks, how much of the time has your physical health or emotional problems interfered with your social activities (like visiting friends, relatives, etc.)?

- ○ All of the time
- ○ Most of the time
- ○ A good bit of the time
- ○ Some of the time
- ○ A little bit of the time
- ○ None of the time

Health Counts 2024 is funded by Brighton & Hove City Council Public Health Department

Overall, how anxious did you feel yesterday? Where 0 is 'not anxious' and 10 is 'completely anxious'
(Move the slider to select)

	Not anxious						Completely anxious				
	0	1	2	3	4	5	6	7	8	9	10

The next questions are about self-harm and suicidal thoughts. Your responses are **confidential** and **anonymous** - you cannot be identified. If you prefer not to answer, you can skip these questions.

In the last 12 months, have you deliberately harmed yourself in any way but not with the intention of killing yourself?

- ◯ Yes
- ◯ No
- ◯ Prefer not to say

In the last 12 months, have you thought of taking your life, even though you would not actually do it?

- ◯ Yes
- ◯ No
- ◯ Prefer not to say

Have you ever made an attempt to take your life?

- ◯ Yes
- ◯ No
- ◯ Prefer not to say

Display This Question:
If Suicide attempt ever = Yes

Was this in the last 12 months?

- ◯ Yes
- ◯ No
- ◯ Prefer not to say

Health Counts 2024 is funded by Brighton & Hove City Council Public Health Department

Help is available for anyone who self-harms or thinks about self-harm, as well as their friends and family. If you have suicidal thoughts or are supporting someone else, the Staying Safe website provides information on how to make a safety plan. You can also speak to your GP or access a free support line such as:

- **Mental Health Line** - 0800 0309 500 – Free 24/7 support
- **Samaritans -** Call 116 123 - Free 24/7 support
- Text "**SHOUT**" to 85258 to contact the Shout Crisis Text Line
- **Mind -** 0300 123 3393 or text 86463 (9am to 6pm on weekdays)

End of Block: Mental health and wellbeing

Start of Block: Smoking & vaping

The next few questions will ask you about **smoking** tobacco. We will ask about **vaping** separately.

Thinking about **smoking tobacco** (cigarettes, roll-ups, cigars, pipes or any other form of smoking such as bidis, kreteks, shisha pipes), which of the following best describes you?

- ◯ I smoke daily
- ◯ I smoke occasionally
- ◯ I used to smoke daily but don't smoke at all now
- ◯ I used to smoke occasionally but don't smoke at all now
- ◯ I have tried smoking but don't smoke now
- ◯ I have never smoked

Display This Question:
If Smoking status = I smoke daily
Or Smoking status = I smoke occasionally

About how many times per day do you smoke (or use tobacco) on average?

____

Do you consume tobacco in any other way?

- ◯ Yes
- ◯ No

Health Counts 2024 is funded by Brighton & Hove City Council Public Health Department

Display This Question:
If Other forms of tobacco consumption = Yes

Which? (Tick all that apply)

- ☐ I use chewing tobacco e.g. paan
- ☐ I use snuff (dry or moist)
- ☐ Other (please specify) ____

Display This Question:
If Smoking status = I smoke daily
Or Smoking status = I smoke occasionally

Do you usually smoke or use tobacco within five minutes of waking up?

- ○ Yes
- ○ No

Display This Question:
If Smoking status = I smoke daily
Or Smoking status = I smoke occasionally

In the last 12 months have you tried to give up smoking?

- ○ Yes
- ○ No

Display This Question:
If Recent quit attempt = Yes

For how long did you give up smoking?

- ○ Less than 4 weeks
- ○ Between 4 weeks and 12 weeks
- ○ More than 12 weeks
- ○ Prefer not to say

Does anyone who lives with you smoke? (inside or outside your home)

- ○ Yes
- ○ No

Health Counts 2024 is funded by Brighton & Hove City Council Public Health Department

Thinking now about vaping, which of the following best describes you?

- I have not tried vaping
- I used to vape but don't any more
- I have tried vaping but don't vape regularly
- I vape occasionally
- I vape every day

End of Block: Smoking & vaping

Start of Block: Alcohol consumption

How often do you have a drink containing alcohol?

- Every day or almost every day
- 4 to 6 times per week
- 2 to 3 times per week
- 2 to 4 times per month
- Monthly or less
- Never

Display This Question:
If Alcohol frequency = Every day or almost every day
Or Alcohol frequency = 4 to 6 times per week
Or Alcohol frequency = 2 to 3 times per week
Or Alcohol frequency = 2 to 4 times per month
Or Alcohol frequency = Monthly or less

[image alcohol unit reference]

How many units of alcohol do you drink on a typical day when you are drinking?

- 1 to 2 units
- 3 to 4 units
- 5 to 6 units
- 7 to 9 units
- 10 or more units

Health Counts 2024 is funded by Brighton & Hove City Council Public Health Department

Display This Question:
If Alcohol frequency = Every day or almost every day
Or Alcohol frequency = 4 to 6 times per week
Or Alcohol frequency = 2 to 3 times per week
Or Alcohol frequency = 2 to 4 times per month
Or Alcohol frequency = Monthly or less

How often have you drunk six or more units on one occasion in the last year?

- ◯ Daily or almost daily
- ◯ Weekly
- ◯ Monthly
- ◯ Less than monthly
- ◯ Never

Display This Question:
If Alcohol frequency = Every day or almost every day
OR Alcohol frequency = 4 to 6 times per week
OR Alcohol frequency = 2 to 3 times per week
OR Alcohol frequency = 2 to 4 times per month
OR Alcohol frequency = Monthly or less

In the last 12 months have you tried to cut down on the amount of alcohol you drink?

- ◯ Yes
- ◯ No

End of Block: Alcohol consumption

Start of Block: Gambling

Do you gamble, even if only occasionally?
By gambling we mean spending money on activities such as lotteries, betting, casino games, fruit/slot machines, and private betting with friends

- ◯ Yes
- ◯ No
- ◯ Prefer not to say

Health Counts 2024 is funded by Brighton & Hove City Council Public Health Department

Display This Question:
If Gambling participation = Yes

Thinking about your **own** gambling, how often in the last 12 months has your own gambling led you to:

	Very often	Fairly often	Occasionally	Never	Prefer not to say
Reduce or cut back your spending on everyday items e.g. food, bills, clothes?	○	○	○	○	○
Use savings or borrow money? e.g. from family/friends, credit cards, overdrafts/loans, money lenders?	○	○	○	○	○
Experience conflict or arguments with friends, family and/or work colleagues?	○	○	○	○	○
Feel isolated from other people, left out or feel completely alone?	○	○	○	○	○
Lie to family, or others, to hide the extent of your gambling?	○	○	○	○	○
Be absent or perform poorly at work or study?	○	○	○	○	○

Does anyone you are close to gamble, even if only occasionally? (Tick all that apply)

☐ ⊗No

☐ Yes, a partner/spouse

☐ Yes, another family member (that is not your partner/spouse)

☐ Yes, a friend

☐ Yes, someone else

☐ ⊗Prefer not to say

Display This Question:
If Gambling-close to = Yes, a partner/spouse
Or Gambling-close to = Yes, another family member (that is not your partner/spouse)
Or Gambling-close to = Yes, a friend
Or Gambling-close to = Yes, someone else

Do you live with this person/any of these people?

- ◯ Yes
- ◯ No
- ◯ Prefer not to say

Display This Question:
If Gambling-close to = Yes, a partner/spouse
Or Gambling-close to = Yes, another family member (that is not your partner/spouse)
Or Gambling-close to = Yes, a friend
Or Gambling-close to = Yes, someone else

This question is about the impact that **someone else's** gambling may have had on you, whether you live with them or not. In the last 12 months, how often has someone else's gambling led you to:

	Very often	Fairly often	Occasionally	Never	Prefer not to say
Reduce or cut back your spending on everyday items e.g. food, bills, clothes?	◯	◯	◯	◯	◯
Use your savings or increase your use of credit such as credit cards, overdrafts and loans?	◯	◯	◯	◯	◯
Experience conflict or arguments with friends, family and/or work colleagues?	◯	◯	◯	◯	◯
Feel isolated from other people, left out or feel completely alone?	◯	◯	◯	◯	◯
Lie to family, or others, to hide the extent of someone else's gambling?	◯	◯	◯	◯	◯
Be absent or perform poorly at work or study?	◯	◯	◯	◯	◯

End of Block: Gambling

Start of Block: Drugs

In the last 12 months, how often (if at all), have you taken drugs that were **not** prescribed for you and were **not** available at a chemist or pharmacy?

- ○ Daily or almost daily
- ○ Weekly
- ○ Monthly
- ○ Less than monthly
- ○ Never
- ○ Prefer not to say

Display This Question:
If Use of drugs, last year = Daily or almost daily
Or Use of drugs, last year = Weekly
Or Use of drugs, last year = Monthly
Or Use of drugs, last year = Less than monthly

What drugs have you used within the last 12 months? (Tick all that apply)
Please **do not** include any drugs that were prescribed for you

- ☐ Heroin or other opiates
- ☐ Crack cocaine
- ☐ Cocaine
- ☐ Methamphetamine
- ☐ Benzodiazepines e.g. Valium, Xanax
- ☐ Cannabis
- ☐ Other non-opiates e.g. ecstasy, spice
- ☐ Other (please specify) ____
- ☐ ⊗Prefer not to say

End of Block: Drugs

Health Counts 2024 is funded by Brighton & Hove City Council Public Health Department

Start of Block: Sexual health

In the last 12 months, how many people have you had sex with? Estimate if you can't say exactly.

Please include people you have had any kind of sex with (vaginal, oral, anal)____

Display This Question:
If If Number of partners, last year Text Response Is Greater Than 0

How many of these were new partners, who you had sex with for the first time in the last year? ________________________________

Estimate if you can't say exactly.

Display This Question:
If If Number of new partners, last year Text Response Is Greater Than 0

How many of these new partners were...
Men : ________
Women : ________
Transgender : ________
Non-binary : ________
Total : ________

Display This Question:
If If Number of new partners, last year Text Response Is Greater Than 0

When you have sex with a new partner for the first time, do you use a condom?

- ◯ Every time
- ◯ Sometimes
- ◯ Never
- ◯ Not applicable
- ◯ Prefer not to say

Have you ever had a test for HIV?

- ◯ Yes
- ◯ No
- ◯ Not sure
- ◯ Prefer not to say

Health Counts 2024 is funded by Brighton & Hove City Council Public Health Department

Display This Question:
If HIV test ever = Yes

When was your most recent HIV test?

- In the last three months
- Between 3 months and up to 1 year ago
- Over 1 year and up to 5 years ago
- More than 5 years ago
- Prefer not to say

PrEP (pre-exposure prophylaxis) can reduce your chance of getting HIV from sex or injection drug use. When taken as prescribed, PrEP is highly effective for preventing HIV. Have you heard of PrEP?

- Yes
- No
- Not sure

Display This Question:
If PreP awareness = Yes

Have you used PrEP?

- No
- Yes, currently
- Yes, in the past
- Prefer not to say

End of Block: Sexual health

Health Counts 2024 is funded by Brighton & Hove City Council Public Health Department

Start of Block: Diet, height and teeth

The next questions will ask about your **diet**, **weight**, and your **teeth**.

How many portions of fruit and vegetables do you eat in a typical day?

1 portion of fruit = a handful of grapes, an orange, a glass of fruit juice, or a tablespoon of dried fruit
1 portion of vegetables = 3 heaped tablespoons of carrots, a side salad, or 2 spears of broccoli

	None	1	2	3	4	5	More than 5
Portions of fruit?	○	○	○	○	○	○	○
Portions of vegetables? (do not include potatoes)	○	○	○	○	○	○	○

Which of the following best describes you?

○ I am underweight

○ I am about the right weight

○ I am a little overweight

○ I am very overweight

○ I am not sure about my weight

We will now ask you about your height and weight. Do you prefer to answer using metric (cm/kg) or imperial (feet/inches and stones/pounds)?

○ Metric

○ Imperial

Display This Question:
If BMI = Metric

Height: How tall are you in centimetres (cm)?

____

Display This Question:
If BMI = Metric

Weight: How much do you weigh in kilogrammes (kg)?

____

Health Counts 2024 is funded by Brighton & Hove City Council Public Health Department

Display This Question:
If BMI = Imperial

Height: How tall are you in feet and inches?

Feet	Inches
--------------	--------------

Display This Question:
If BMI = Imperial

Weight: How much do you weigh in stones and pounds?

Stones	Pounds
--------------	--------------

End of Block: Diet, height and teeth

Start of Block: Oral health

How often do you clean your teeth?

- ◯ More than twice a day
- ◯ Twice a day
- ◯ Once a day
- ◯ Less than once a day
- ◯ Never

Health Counts 2024 is funded by Brighton & Hove City Council Public Health Department

Where do you go for help if you have problems with your teeth or gums? (Tick all that apply)

- [ ] Accident & Emergency (hospital)
- [ ] Dentist
- [ ] Emergency Dental Service
- [ ] GP
- [ ] Hygienist
- [ ] Pharmacy
- [ ] ⊗I don't go anywhere for help
- [ ] Other (please specify) ____

How often do you go to the dentist?

- ○ At least once every six months
- ○ At least once every year
- ○ At least once every two years
- ○ Less frequently than every two years
- ○ I never go to the dentist

Health Counts 2024 is funded by Brighton & Hove City Council Public Health Department

Display This Question:
If Frequency dental visit = I never go to the dentist

Why do you not go to the dentist? (Tick all that apply)

- ☐ I have been unable to register with an NHS dentist
- ☐ I have been unable to afford to see a private dentist
- ☐ I have been unable to travel to the nearest dentist
- ☐ I have fear/ anxiety about seeing a dentist
- ☐ I have not felt that I needed to see a dentist
- ☐ My usual dental practice has closed
- ☐ Other (please specify) ____
- ☐ ⊗Don't know
- ☐ ⊗Prefer not to say

Display This Question:
If Frequency dental visit = At least once every six months
Or Frequency dental visit = At least once every year
Or Frequency dental visit = At least once every two years
Or Frequency dental visit = Less frequently than every two years

Generally, why do you go to the dentist?

- ○ For a regular check up
- ○ For an occasional check up
- ○ Only when I have trouble with my mouth, teeth or dentures
- ○ Other (please specify) ____

End of Block: Oral health

Health Counts 2024 is funded by Brighton & Hove City Council Public Health Department

Start of Block: Housing and cost of living

What is your housing situation now?

- ◯ Own my own home (with/without a mortgage)
- ◯ Part own and part-rent (shared ownership)
- ◯ Rent my home (housing association or local council)
- ◯ Rent my home (private landlord)
- ◯ Live rent-free with job, shop or other business
- ◯ Live with parents/family
- ◯ Live in temporary or emergency accommodation e.g. shelter, sofa surfing, bed & breakfast accommodation
- ◯ Squatting
- ◯ Other (please specify) ____

How worried are you about your housing conditions? e.g. damp, cold, leaks etc.

- ◯ Very worried
- ◯ Fairly worried
- ◯ Not very worried
- ◯ Not worried at all
- ◯ Don’t know

Health Counts 2024 is funded by Brighton & Hove City Council Public Health Department

Which of these, if any, are you doing because of the increases in the cost of living? (Tick all that apply)

- ☐ Shopping around more
- ☐ Spending less on food
- ☐ Reducing leisure activities
- ☐ Reducing non-essential journeys
- ☐ Using less fuel at home
- ☐ Making energy efficiency improvements to my home
- ☐ Using my savings
- ☐ Using credit cards, loans or overdrafts more than usual
- ☐ Using support from charities e.g. food banks
- ☐ Other things (please specify) ____
- ☐ ⊗None of these

End of Block: Housing and cost of living

Start of Block: Your local area

We would now like to ask you about the **area in which you live**, including use of open spaces and access to nature, contact with neighbours, and worries about crime. Please answer every question.

Overall, how satisfied or dissatisfied are you with your local area as a place to live?

- ○ Very satisfied
- ○ Fairly satisfied
- ○ Neither satisfied or dissatisfied
- ○ Fairly dissatisfied
- ○ Very dissatisfied

How strongly do you feel you belong to your immediate neighbourhood?

- ◯ Very strongly
- ◯ Fairly strongly
- ◯ Not very strongly
- ◯ Not at all strongly
- ◯ Don't know

How often do you see or speak to your neighbours?

- ◯ Every day
- ◯ 5 or 6 days a week
- ◯ 3 or 4 days a week
- ◯ Once or twice a week
- ◯ Once or twice a month
- ◯ Once every couple of months
- ◯ Once or twice a year
- ◯ Not at all in the last 12 months

If you were ill in bed and needed help at home, could you ask anyone for help? Help at home means help with domestic tasks such as cooking, cleaning, and making a cup of tea.

- ◯ Yes
- ◯ No
- ◯ Don't know / it depends

Health Counts 2024 is funded by Brighton & Hove City Council Public Health Department

In the last 12 months, how often, on average have you spent free time outside in green and natural spaces?

- ◯ Every day
- ◯ More than twice a week, but not every day
- ◯ Twice a week
- ◯ Once a week
- ◯ Once or twice a month
- ◯ Once every 2 to 3 months
- ◯ Less often
- ◯ Never
- ◯ Don't know
- ◯ Prefer not to say

Health Counts 2024 is funded by Brighton & Hove City Council Public Health Department

Display This Question:
If Access to nature_frequency = Every day
Or Access to nature_frequency = More than twice a week, but not every day
Or Access to nature_frequency = Twice a week
Or Access to nature_frequency = Once a week
Or Access to nature_frequency = Once or twice a month
Or Access to nature_frequency = Once every 2 to 3 months
Or Access to nature_frequency = Less often

Which of the following types of green and natural spaces have you visited during the last 12 months? (Tick all that apply)

- ☐ Urban green space e.g. park, playground
- ☐ Grounds of historic property or country park
- ☐ Allotment or community garden
- ☐ Woodland or forest
- ☐ River, lake or canal
- ☐ Hill, mountain or moorland
- ☐ Beach / other coastline / sea
- ☐ Nature / wildlife reserve
- ☐ Fields / farmland / countryside
- ☐ Another green and natural space (please specify) ____
- ☐ ⊗Don't know
- ☐ ⊗Prefer not to say

How safe do you feel walking alone in your local area within approximately 15 minutes from your home? If you don't walk alone, please answer how safe you would feel if you did.

	Very safe	Fairly safe	A bit unsafe	Very unsafe
In the daytime	○	○	○	○
At night	○	○	○	○

How worried are you about the following?

	Very worried	Fairly worried	Not very worried	Not at all worried	Not applicable
Physical violence against yourself	○	○	○	○	○
Physical violence against a family member	○	○	○	○	○
Being sexually assaulted/raped	○	○	○	○	○
Being targeted by perpetrators of hate crime because of your ethnic heritage or religion or perceived ethnic heritage or religion	○	○	○	○	○
Being targeted by perpetrators of hate crime because of your sexual orientation or perceived orientation	○	○	○	○	○
Being targeted by perpetrators of hate crime because you are trans, non-binary or present as gender divergent	○	○	○	○	○
Being targeted by perpetrators of hate crime because of your disability or assumed disability	○	○	○	○	○
Being targeted by perpetrators of hate crime because of your clothing or visible difference	○	○	○	○	○

Health Counts 2024 is funded by Brighton & Hove City Council Public Health Department

In the past 12 months, have you experienced any of the following? (Tick all that apply)

- ☐ Been insulted or shouted at by a stranger in a public space
- ☐ Experienced catcalls, whistles, unwanted sexual comments or jokes from a stranger in a public space
- ☐ Felt that you were being followed
- ☐ Felt physically threatened by a stranger in a public space
- ☐ ⊗None of the above
- ☐ ⊗Don't know
- ☐ ⊗Prefer not to say

End of Block: Your local area

Start of Block: Some final questions about you...

Some **final questions** about you... these last questions are important to help us understand more about you and your responses. Please answer every question.

Which best describes your gender?

- ○ Female
- ○ Intersex
- ○ Male
- ○ Non-binary
- ○ Prefer to self-describe (please specify) ____
- ○ Prefer not to say

Are you trans or do you have a trans history?

Trans or transgender is an umbrella term for people whose identity differs from what is typically associated with the sex they were assigned at birth

- ○ Yes
- ○ No
- ○ Prefer not to say

Health Counts 2024 is funded by Brighton & Hove City Council Public Health Department

Which of the following best describes your sexual orientation?

- ◯ Asexual
- ◯ Bisexual or bi
- ◯ Gay or lesbian
- ◯ Heterosexual or 'straight'
- ◯ Queer
- ◯ If you prefer to use another term, please specify ____
- ◯ Prefer not to say

What is your ethnic group?

- ◯ Asian or Asian British
- ◯ Black, Black British, Caribbean or African
- ◯ Mixed / Multiple ethnic groups
- ◯ Other ethnic group
- ◯ White

Display This Question:
If Ethnicity = Asian or Asian British

Are you...

- ◯ Indian
- ◯ Pakistani
- ◯ Bangladeshi
- ◯ Chinese
- ◯ Any other Asian background (please specify) ____

Health Counts 2024 is funded by Brighton & Hove City Council Public Health Department

Display This Question:
If Ethnicity = Black, Black British, Caribbean or African

Are you...

- ◯ Caribbean
- ◯ African background (please specify) ____
- ◯ Any other Black, Black British, or Caribbean background (please specify) ____

Display This Question:
If Ethnicity = Mixed / Multiple ethnic groups

Are you...

- ◯ White and Black Caribbean
- ◯ White and Black African
- ◯ White and Asian
- ◯ Any other Mixed / Multiple ethnic background (please specify) ____

Display This Question:
If Ethnicity = Other ethnic group

Are you...

- ◯ Arab
- ◯ Any other ethnic group (please specify) ____

Display This Question:
If Ethnicity = White

Are you...

- ◯ English, Welsh, Scottish, Northern Irish or British
- ◯ Irish
- ◯ Gypsy or Irish Traveller
- ◯ Roma
- ◯ Any other White background (please specify) ____

Health Counts 2024 is funded by Brighton & Hove City Council Public Health Department

What is your religion?

- ○ Buddhist
- ○ Christian including Church of England, Catholic, Protestant and all other Christian denominations
- ○ Hindu
- ○ Jewish
- ○ Muslim
- ○ No religion
- ○ Sikh
- ○ Any other religion (please specify) ____
- ○ Prefer not to say

Do you look after, or give any help or support to, anyone because they have long-term physical or mental health conditions or illnesses, or problems related to old age?
Please exclude anything you do as part of your paid employment.

- ○ No
- ○ Yes, 9 hours a week or less
- ○ Yes, 10 to 19 hours a week
- ○ Yes, 20 to 34 hours a week
- ○ Yes, 35 to 49 hours a week
- ○ Yes, 50 or more hours a week
- ○ Prefer not to say

Health Counts 2024 is funded by Brighton & Hove City Council Public Health Department

Display This Question:
If Carer status = Yes, 9 hours a week or less
Or Carer status = Yes, 10 to 19 hours a week
Or Carer status = Yes, 20 to 34 hours a week
Or Carer status = Yes, 35 to 49 hours a week
Or Carer status = Yes, 50 or more hours a week

Who do you look after, or give help or support to? (Tick all that apply)

- ☐ Parent
- ☐ Partner/spouse
- ☐ Child with special needs
- ☐ Friend
- ☐ Other family member
- ☐ Other (please specify) ____

Did you *ever* live in care when you were a child or young person?

Living in care means being in the care of the local authority or on a care order, for more than 24 hours and living away from your parents e.g. with foster parents, other family members, residential children's home, or residential setting like a school or secure unit.

- ○ Yes
- ○ No
- ○ Prefer not to say

Are you a refugee or asylum seeker?

Please remember, all responses are anonymous and cannot be traced back to you

- ○ No
- ○ Yes, I have been recognised as a refugee by the UK government
- ○ Yes, I am seeking asylum
- ○ Prefer not to say

Health Counts 2024 is funded by Brighton & Hove City Council Public Health Department

Have you previously served in the UK Armed Forces? (Tick all that apply)

This includes reservists or part-time service, such as the Territorial Army

- ☐ Yes, previously served in Regular Armed Forces
- ☐ Yes, previously served in Reserve Armed Forces
- ☐ ⊗No
- ☐ ⊗Prefer not to say

Are you currently serving in the UK Armed Forces? (Tick all that apply)

- ☐ Yes in the Regular Armed Forces
- ☐ Yes in the Reserve Armed Forces
- ☐ ⊗No
- ☐ ⊗Prefer not to say

Are you part of an Armed Forces Family? Is anyone in your household in the Army, Navy or Airforce? Or have been in the past?

- ○ Yes
- ○ No
- ○ Prefer not to say

End of Block: Some final questions about you...

Start of Block: And finally...

Prize Draw Entry
As a thank-you for completing the survey, you can enter a prize draw to win **£100 voucher** by providing your contact details. These are stored separately from your answers and will ***only*** be used for the prize draw. Once the draw is complete and a winner selected randomly, all contact details will be ***deleted***. If you have won, you will be notified in May 2024. If you would like to enter the prize draw please enter your email address or other contact information.

____

Please now click **SUBMIT**

You will be re-directed back to the Health Counts 2024 home page where you can find information about organisations that can support you

Thank you for completing this survey

End of Block: And finally...
